# Pictograms for safer medication handling by health care workers: a validation study in nursing students in Poland

**DOI:** 10.1186/s12913-022-08029-8

**Published:** 2022-05-13

**Authors:** Piotr Merks, Regis Vaillancourt, Damien Roux, Rafał Gierczyński, Grzegorz Juszczyk, Katarzyna Plagens-Rotman, Urszula Religioni, Jameason Cameron, Mike Zender

**Affiliations:** 1 Faculty of Medicine, Cardinal Stefan Wyszyński University in Warsaw, Warsaw, Poland; 2 Children’s Hospital of Eastern Ontario, CHEO, 401 Smyth Rd, Ottawa, Canada; 3 Division of Epidemiological and Environmental Safety, National Institute of Public Health NIH - National Research Institute, Warsaw, Poland; 4 Department of Public Health, Medical University of Warsaw, Warsaw, Poland; 5 Hipolit Cegielski State University of Applied Sciences, Gniezno, Poland; 6 School of Public Health, Centre of Postgraduate Medical Education of Warsaw, Warsaw, Poland; 7 School of Design, University of Cincinnati, Cincinnati, OH USA

**Keywords:** Pictograms, Medication safety, Safe medication handling, Medication error, Pharmaceutical care

## Abstract

**Background and objective:**

Medication use often causes errors that are dangerous to the health of patients. Previous studies indicate that the use of pharmaceutical pictograms can effectively reduce medication errors. The purpose of this study was to determine the comprehensibility, representativeness, and recall rate of nine medication safety pictograms in a sample of nursing students in Poland in order to validate these images.

**Methods:**

A pictogram validation study was conducted in two phases among nursing students at the Hipolit Cegielski State University of Applied Sciences, Gniezno, Poland.

All experimental protocols were approved by the Children's Hospital of Eastern Ontario Research Ethics Board (REB Protocol No: 19/122X). All methods were carried out in accordance with relevant guidelines and regulations.

In phase 1, the participants' first exposure to the pictograms, the students were asked to guess the meaning of the pictograms without any additional information in order to assess the pictograms' comprehensibility. To be considered valid, according to ISO standards, the pictograms had to be correctly understood by at least 66.7% of participants. After testing all pictograms, students were given explanations and meanings of the pictograms and asked to rate the representativeness of pictograms. To do so, participants were asked to select a number on a seven-point Likert-style scale to indicate the perceived strength of the relationship between the pictogram and its intended meaning for each pictogram. To be considered valid, a pictogram had to be rated at least five on this scale by at least 66.7% of participants. Phase 2 took place four weeks later, during which recall of the intended meaning and representativeness were assessed following the same procedure.

**Results:**

A total of 66 third-year nursing students participated in both phases. In phase 1, of the nine pictograms, six met ISO requirements for comprehensibility and seven met ISO requirements for representativeness. In phase 2, all nine pictograms were correctly understood and rated at least 5 by at least 66.7% of participants. Therefore, all nine pictograms are considered valid.

**Conclusions:**

The nine medication safety pictograms can be deployed, but must be combined with training and a written hazard statement to improve comprehension.

## Background

For drug therapy to be optimal, the right patient should receive the right dose of the right drug, in the right way, at the right time [1]. Unfortunately, these conditions are often not met in real-life situations, leading to a high number of medication errors [2, 3]. These errors can be clinically inconsequential or be seriously harmful to patients resulting in hospitalization, life-threatening situations, disability, or even death [1–4]. Nevertheless, they also represent an economic burden [5, 6], a source of trauma for the staff and weaken the patient-healthcare provider relationship [4]. Despite this, they are still widespread [7], especially during prescription and administration [2, 3]. The medication errors occuremainly due to deficiencies in training, overwork of the healthcare workers, lack of communication, policies inadequate to the actual practice, lack of consistency in procedures or the absence of pharmaceutical follow-up [4].

In recent years, medication error management has undergone a positive evolution from a blame-based approach to a systems approach that seeks to identify and address the underlying causes of medication error [8]. Thus, the focus is now on the provision of protocols, tools and resources designed to help decrease medication administration errors such as bar-coding systems, weight-based dosing, double- or triple-checking systems, increased pharmacist involvement, avoiding abbreviations, etc.[3–5] One of the key points identified to reduce the risk of medication errors is to provide access to critical characteristics of drug during administration, however, the time constraints faced by healthcare professionals require these reminder systems to be concise [4].

Thus, pictograms could be an interesting alternative because of their visual impact and their ability to convey information in a concise way regardless of language skills [9]. According to various cognitive theories [10–12], pictograms can increase recall of instructions, especially when combined with training. The cautionary pictograms of the Globally Harmonized System for the classification and labelling of chemicals (GHS) have already been used by the Workplace Hazardous Materials Information System (WHMIS) to increase workplace safety during the handling of chemicals [13]. A similar tool could improve the handling of medication by healthcare professionals, especially during the administration phase [14, 15]. However, the interpretation of a pictogram is person-dependent and therefore likely to be different according to culture, profession, age, etc. Thus, after the design and implementation of a pictogram, it is essential to complete a validation step of the pictogram understanding by the target population [16].

In a previous study conducted in Canada in collaboration with the Institute for Safe Medication Practices (ISMP), researchers have identified key medication safety categories that could benefit from the implementation of safety pictograms, including look-alike, sound-alike drugs, concentrated electrolyte solutions, and medications that have a high incidence of dosage/calculation error [17]. Pictograms for each of the nine key medication safety issues have been developed by a panel of international healthcare workers. In their study, Vaillancourt et al.[18] took a first step towards validation of these nine pictograms in a sample of pharmacy students in Canada. These students were able to guess the meaning of four of the nine pictograms and to recall the meanings of seven of the pictograms. These results suggest that at least two of the medication safety pictograms are not easily understood. However, pharmacists and therefore pharmacy students may be less aware of medication administration safety because they do not administer medications.

To further test the medication safety pictograms, nursing students in their final stages of study appear to be a good population. Indeed, as future nurses, they will be the health care professionals most often responsible for administering medications in hospitals, they have already completed hospital internships and received the majority of their education. Furthermore, medication safety pictograms must be understood by the most junior and least experienced health care professionals who administer medications because they are at risk of making errors [4].

This research project aimed to determine the comprehensibility, representativeness, and rate of recall for nine medication safety pictograms in a sample of nursing students in Poland in order to validate these images.

## Methods

This study was conducted by Cardinal Stefan Wyszyński University, Warsaw, Poland among nursing students at the Hipolit Cegielski State University of Applied Sciences, Gniezno, Poland.

### Study design/data collection

**Fig. 1:**
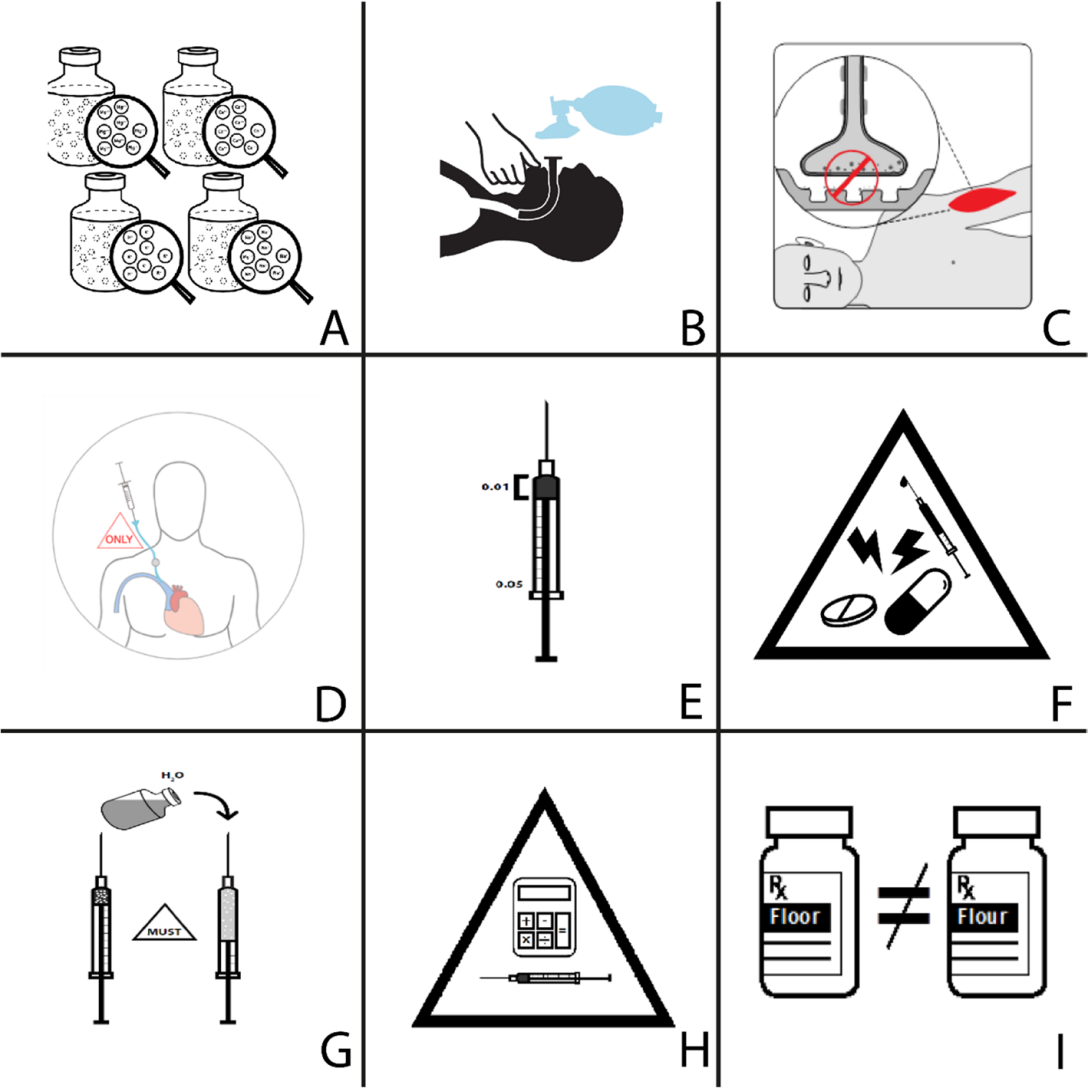
Pictograms and their intended meanings. **A** Concentrated electrolyte formulation. **B** Medication that requires airway management before administration. **C**, Neuromuscular blocking agent. **D**, Medication that can be given only via central line. **E**, Medication that has a low volume dose. **F** Medication with a significant risk of harm if administered improperly. **G** Medication that must always be diluted before administration. **H**, Medication that has a high incidence of calculation/dosage error. **I** Medication names that look alike and sound alike *Pictograms © 2016 by Régis Vaillancourt, The CHEO Research Institute, and Mike P Zender; reproduced with permission

There were 2 phases to the validation. The first phase began with a presentation of the project given by the research assistant (RA). The comprehensibility test was conducted by showing pictograms (Fig. 1) on a PowerPoint presentation projected on a large screen and asking participants to guess the meaning of the pictogram without any additional information provided. After all the pictograms were tested, the students were given explanations and meanings of the pictograms and asked to rate the strength of the relationship between each pictogram and its intended meaning. The session was led by a member of the research team and not by a Hipolit Cegielski staff member to avoid any perception of coercion.

The second phase took place four weeks later to test long term recall. The same group of participants was asked to recall the meanings of the nine original medication safety pictograms, evaluate the relationship between each pictogram and its intended meaning, following the same procedure as during the first phase.

Administration of surveys and data collection was done using a paper and pencil questionnaire. Data collection was performed by nurses with PhD degree among students at the Hipolit Cegielski University, which is training nurses. Data was then entered into an Excel database.

### Participants

Students from the nursing program at Hipolit Cegielski State University of Applied Sciences were invited to participate in the study during class time in a course chosen by the Chair of Nursing Studies. Those who consent to participate remained in the classroom to complete the study. Nurses already working are supposed to understand the pictograms more easily than students because of their experience, so they were excluded in order to avoid biasing the results.

## Measurements

### Demographics

Participants were asked about gender, language, and year of study.

### Comprehension of pictograms

Pictogram comprehension is defined as "the process of interpreting words or pictures to understand their collective meaning" [19, 20], and was determined by assessing the pictogram transparency and translucency. We used the standards for the testing of safety signs from the International Organization for Standardization (ISO). The ISO 9186–1 criteria for acceptability in the testing of safety signs are that the percentage of respondents comprehending the sign must be 66.7% or more [21].

### Transparency

Transparency is the guessability of the meaning of a picture or illustration when the signification is unknown by participants [19, 20]. Transparency was assessed by presenting the participant with pictograms and asked them "In the context of medication administration, what do you think the image means?". Responses were assigned as "correct" or "incorrect". To be considered "correct," the guessed meaning had to match the intended meaning.

### Process of understanding

According to the semiotic triangle of Peirce, the pictograms are composed of three dimensions: a representamen, an interpretant and a referent [22]. The representamen -or image- designates the components of the image, it is the perceptible face of the sign. The interpretant -or concept- designates the perspective under which the image is apprehended by the observer, it is the mental concept associated with the image. The referent -or object- is the conceptual reality associated with the pictogram, or the intended meaning. To correctly understand the intended meaning of a pictogram, it is necessary to correctly understand the image itself, then to associate it with the correct concept, and finally to correctly relate this concept to the situation. Transparency previously allowed us to assess comprehension of the intended meaning, which is the final step in comprehension. In order to evaluate the whole process of pictogram comprehension and to identify the step that may be problematic, we assessed image and concept comprehension. Based on the participant's response to the transparency question, we determined whether the components of the image were correctly understood in the context of medication safety and rated the responses as "correct" or "incorrect". The same procedure was then applied for the concept understanding.

### Translucency

Translucency evaluates the strength of the relationship between the picture and its intended meaning [20]. Translucency was assessed by presenting the participant each of the pictograms along with the text explaining the intended meaning of the pictogram. The package was separated from the transparency questionnaire package to reduce the likelihood that a participant will inadvertently see the text description of the pictograms. For each pictogram, the participant was asked to select a number on a seven-point, Likert-style scale to indicate the perceived strength of the relationship between the pictogram and its referent. Scores gone from "1 = no relationship between pictogram and its meaning" to "7 = strong relationship between pictogram and its meaning". To be considered as valid, a pictogram must be rated at least a five on this scale by at least 66.7% of participants.

### Statistical analysis

McNemar association tests were performed to compare the transparency, image and concept comprehension and translucency results obtained in phase 1 with the results obtained in phase 2. The McNemar test was chosen for the analysis of correlated or dependent dichotomous variables (Yes or No answers for understanding the pictorgram) with a pre-post testing design. (McNemar, Q. (1947). Note on the sampling error of the difference between correlated proportions or percentages. Psychometrika, 12(2), 153–157. https://doi.org/10.1007/bf02295996).

## Results

### Demographics

A total of 66 participants were included in this study. Participants were all third-year nursing students with Polish as mother language. There were 57 (86.4%) women and 9 (13.6%) men. All the 66 first phase participants also participated in the second phase.

### Phase 1: First exposure to the pictograms

#### Transparency

Six of the nine pictograms presented were correctly guessed by at least 66.7% of the participants, thus meeting the requirements of the ISO standard (Table 1). The three pictograms correctly guessed by less than 66.7% of participants were "Concentrated electrolyte formulation" (31.8%), "Neuromuscular blocking agent" (56.4%), and "Drug requiring airway management before administration" (57.6%).

**Table 1:** Transparency of 9 medication safety pictograms in phase 1 and phase 2 among 66 nursing students

Intended meaning	Phase 1 Correct guesses (*n* = 66)	Phase 2 Correct guesses (*n* = 66)
	**No. (%)**	**No. (%)**
**Concentrated electrolyte formulation**	**21 (31.8%)**	**63 (95.5%)**
**Medication that requires airway management before administration**	**38 (57.6%)**	**66 (100%)**
**Neuromuscular blocking agent**	**41 (62.1%)**	**66 (100%)**
**Medication that can be given only via central line**	**56 (84.8%)**	**66 (100%)**
**Medication that has a low volume dose**	**59 (89.4%)**	**66 (100%)**
**Medication with a significant risk of harm if administered improperly**	**60 (90.9%)**	**66 (100%)**
**Medication that must always be diluted before administration**	**60 (90.9%)**	**66 (100%)**
**Medication that has a high incidence of calculation/dosage error**	**64 (97.0%)**	**66 (100%)**
**Medication names that look alike and sound alike**	**66 (100%)**	**66 (100%)**
**Total correct guesses**	**465 (78.3%)**	**591 (99.5%)**

The most common incorrect guess for the "Concentrated electrolyte formulation" pictogram was "Drug that contains electrolyte" (34 [51.5%]). Concerning the pictogram depicting "Drug requiring airway management before administration", the most frequent incorrect answer was "Drug administered through the airway, inserting an oropharyngeal airway" (25 [37.9%]). All the participants who misinterpreted the pictogram "Neuromuscular blocking agent" (25 [37.9%]) assigned it the meaning "Drug administered intramuscularly".

#### Process of understanding

The images and concepts of the six pictograms that met the transparency requirements were correctly understood. For the pictogram representing "Concentrated electrolyte formulation", 62 (93.9%) participants correctly understood the image but only 22 (33.3%) accurately identified the associated medication safety concept. For the pictogram depicting "Medication requiring airway management before administration", 66 (100.0%) participants correctly understood the image but only 39 (59.1%) associated it with the correct concept. The image and concept of the pictogram "Neuromuscular blocking agent" were correctly understood by 41 (62.1%) participants.

#### Translucency

Of the nine pictograms, seven were considered to have a strong relationship with the intended meaning (score ≥ 5) by at least 66.7% of the participants (*Table **2*). However, the pictograms representing "Medication that requires airway management before administration" (50.0%) and "Neuromuscular blocking agent" (65.2%) did not meet standard ISO requirements.

**Table 2:** Translucency of 9 medication safety pictograms in phase 1 and phase 2 among 66 nursing students

Intended meaning	Phase 1 Correct guesses (*n* = 66)	Phase 2 Correct guesses (*n* = 66)
	**No. (%)**	**No. (%)**
**Concentrated electrolyte formulation**	**56 (84.8%)**	**66 (100%)**
**Medication that requires airway management before administration**	**33 (50%)**	**66 (100%)**
**Neuromuscular blocking agent**	**43 (65.2%)**	**65 (98.5%)**
**Medication that can be given only via central line**	**60 (90.9%)**	**66 (100%)**
**Medication that has a low volume dose**	**57 (86.4%)**	**66 (100%)**
**Medication with a significant risk of harm if administered improperly**	**46 (69.7%)**	**65 (98.5%)**
**Medication that must always be diluted before administration**	**59 (89.4%)**	**66 (100%)**
**Medication that has a high incidence of calculation/dosage error**	**50 (75.8%)**	**65 (98.5%)**
**Medication names that look alike and sound alike**	**60 (90.9%)**	**66 (100%)**
**Total translucency scores** $\ge 5$	**464 (78.1%)**	**591 (99.5%)**

### Phase 2: Recall

#### Transparency

During phase 2, all nine pictograms were correctly guessed by at least 66.7% of the participants, thus meeting ISO requirements. In total, pictograms were significantly more guessed in phase 2 (n = 591 [99.5%]) than in phase 1 (*n* = 465 [78.3%]) (*p* < 0.001 by McNemar test) (Table 1).

#### Process of understanding

Since the intended meanings of the nine pictograms were largely correctly identified, the images and concepts of the nine pictograms were correctly understood as well. Participants were more likely to understand the image of the pictograms in phase 2 than in phase 1 (547 [92.1%] in phase 1 versus 594 [100.0%] in phase 2; *p* < 0.001 by McNemar test). During phase 2, they were also more likely to identify the medication safety concept associated with the pictograms than they were in phase 1 (471 [79.3%] in phase 1 versus 591 [99.5%] in phase 2; *p* < 0.001 by McNemar test).

#### Translucency

All the nine pictograms met the standard ISO requirements, being considered to have a strong relationship with the intended meaning (score ≥ 5) by at least 66.7% of participants. In total, pictograms received significantly more scores ≥ 5 in phase 2 than in phase 1 (464 [78.1%] in phase 1 versus 591 [99.5%] in phase 2; *p* < 0.001) (*Table **2*).

## Discussion

Although pictograms can be an effective tool in preventing medication errors during administration, they can have the opposite effect if the message they convey is not clearly perceived. It was therefore important to test the pictograms on students and healthcare professionals responsible for medication administration prior to their implementation. In phase 1, when participants were first presented with the pictograms and without additional information about their meaning, six of the nine pictograms were correctly understood by at least 66.7% of participants. In phase 2, four weeks after the intended meaning of the pictograms was revealed, participants were significantly more able to correctly interpret the pictograms. Indeed, all nine pictograms were correctly understood by almost all participants, which is well above the minimum 66.7% required by the ISO standard. Thus, the three pictograms that did not meet the ISO standard during phase 1 were finally considered as valid during the recall phase. This demonstrates that with training on the meaning of the pictograms, even if brief, these pictograms can be understood and easily memorized by health care professionals, thus be able to convey the intended messages. Representativeness scores also increased significantly in phase 2, reaching a total of 99.5% scores ≥ 5. This indicates that the training not only leads to high recall of the meaning of the pictogram, but also strengthens participants' perception of the relationship between the pictogram and the intended meaning, to the extent that it becomes obvious for them.

The pictograms had previously been submitted to validation test in a sample of Canadian pharmacy students [18]. It is expected that the nursing students have not been exposed to these pictograms as these have not been used in clinical practice. Overall, Polish nursing students were more likely to understand the pictograms than Canadian pharmacy students, suggesting that nursing students' exposure to medication administration enhances their appreciation of pictograms. Indeed, pharmacy students correctly guessed four pictograms in phase 1 and seven out of nine in phase 2. Two ("Concentrated electrolyte formulation" and "Neuromuscular blocking agent") of the three pictograms that did not meet ISO standards in phase 1 among nursing students also did not meet standards among pharmacy students, confirming that the messages conveyed by these two pictograms are particularly complex to understand. However, the "Concentrated electrolyte formulation" pictogram was more likely to be guessed by nurses, suggesting that the hypothesis by Vaillancout et al. that first- and second-year pharmacy students did not comprehend it due to their educational stage was likely. The "Neuromuscular blocking agent" pictogram was similarly guessed by Canadian pharmacy students. The pictogram "Drug requiring airway management" was correctly guessed by 69.3% of pharmacy students compared with 57.6% of nursing students, but this difference could be explained by the different sample size. In phase 2 among pharmacy students, two pictograms, "Concentrated electrolyte formulation" and "Medication with a significant risk of harm if administered improperly", were still correctly interpreted by less than 66.7% of pharmacy students, whereas all pictograms were largely consistent with ISO standards among nursing students. This better understanding of the pictograms by the nursing students may be explained by the fact that they are directly involved in the process of administration, more aware of the issues and therefore more likely to remember them, but also by the fact that they had more years of education than the first- and second-year pharmacy students. However, these comparisons must be taken with the necessary hindsight, as the transparency response raters were different between the two studies and could therefore gave different corrections.

The "Drug that requires airway management" pictogram did not meet the guessability requirements in phase 1. The image was easily understood, but the concept comprehension appeared problematic. This was confirmed during the translucency test with only 50.0% of representativeness scores ≥ 5 and recommendations given by participants that the intended meaning should be adjusted because it did not match the image. This confusion could be addressed by adding text with the pictogram to avoid confusion, along the same model as the WHMIS pictograms [13]. Indeed, WHMIS pictograms include a "signal word" to alert the reader to a potential hazard and a "hazard statement" describing the nature of the hazard. After the recall phase, guessability met ISO requirements with 100% correct guesses and representativeness score ≥ 5, suggesting that training was sufficient to improve the clarity of the concept for participants.

The fact that the pictogram depicting "Neuromuscular blocking agent" did not meet the guessability requirements in phase 1 was due to poor understanding of the image. This was confirmed in the translucency phase with only 65.2% of representativeness scores ≥ 5 and nine out of ten recommendations given by participants reporting that the pictogram was unclear. In contrast, in the recall phase, confusion was no longer made with 100% correct guesses and 98.5% representativeness scores ≥ 5, thus training (during phase 1 of the study) ultimately resulted in correct recall of the intended meaning without need for redesign.

The "Concentrated Electrolyte Formulation" pictogram did not meet the transparency requirement in phase 1. Participants could identify electrolytes, however they did not associate it the concept of high concentration. Nevertheless, the phase 1 translucency results were consistent with ISO standards, suggesting that the addition of a "hazard statement" describing the nature of the hazard should be sufficient to make the pictogram understandable. Alternatively, the more transparent pictogram needs to be proposed.

Although these pictograms were designed in North America, the Polish nursing students were able to understand all of them well enough to meet the ISO requirements in phase 2. Thus, despite differences in culture, representations and language, the pictograms are understandable. This result confirms the relevance of the design of the pictograms to the actual practices and challenges encountered by nurses. It could be interesting to test these pictograms in other countries and with other health professionals administering medication.

## Conclusion

The nine medication safety pictograms can be considered as valid. However, further research is needed to check transparency and the understandability of pictograms in the group of hospital medical workers. It is important to assess the legibility of pictograms in everyday clinical practice, including their size and placement, taking into account the large amount of work, pressure and the rush. Research combining iteration testing of pictogram design as well as pictograms in combination with the hazard statements will be of particular importance.

Since some of these pictograms are complicated to understand, adding a hazard statement with the pictograms would greatly improve their comprehensibility. Nevertheless, it is necessary to associate pictograms with training prior to their implementation in order to facilitate the memorization of their meaning.

## Data Availability

All data are available from the corresponding author.
